# *Seseli tortuosum* L. subsp. *tortuosum* Essential Oils and Their Principal Constituents as Anticancer Agents

**DOI:** 10.3390/plants13050678

**Published:** 2024-02-28

**Authors:** Alessandro Vaglica, Antonella Maggio, Natale Badalamenti, Maurizio Bruno, Marianna Lauricella, Chiara Occhipinti, Antonella D’Anneo

**Affiliations:** 1 Department of Biological, Chemical and Pharmaceutical Sciences and Technologies (STEBICEF), University of Palermo, Viale delle Scienze, Ed. 17, 90128 Palermo, Italy; alessandro.vaglica@unipa.it (A.V.); antonella.maggio@unipa.it (A.M.); natale.badalamenti@unipa.it (N.B.); 2 NBFC—National Biodiversity Future Center, 90133 Palermo, Italy; 3 Department of Biomedicine, Neurosciences and Advanced Diagnostics (BIND), Institute of Biochemistry, University of Palermo, 90127 Palermo, Italy; marianna.lauricella@unipa.it; 4 Laboratory of Biochemistry, Department of Biological, Chemical and Pharmaceutical Sciences and Technologies (STEBICEF), University of Palermo, 90127 Palermo, Italy; chiara.occhipinti@unipa.it (C.O.); antonella.danneo@unipa.it (A.D.)

**Keywords:** apiaceae, *p*-cymene, *β*-ocimene, colon cancer cells, GC-MS, *Seseli tortuosum* L. subsp. *tortuosum*

## Abstract

*Seseli tortuosum* L. subsp. *tortuosum*, belonging to the Apiaceae family, is a species that grows in Europe, mainly in the Mediterranean regions. The history of its application in traditional medicine highlights its various biological properties. Trying to explore the phytochemistry and pharmacological aspects of this species, the essential oils (EOs) extracted from flowers, stems, and roots of a locally wild accession, never previously investigated, growing in Sicily, Italy, were investigated. The chemical composition of all EOs, obtained by the hydrodistillation method, was evaluated by GC-MS. The most abundant class of all investigated samples was that of monoterpene hydrocarbons (79.98–91.21%) with *p*-cymene, *α*-pinene, *β*-pinene, and *β*-ocimene as major compounds. These EOs, and their main components, were tested for their possible anticancer activity. Obtained data provided evidence that among the different EOs tested, at the dose of 100 μg/mL, those extracted from stems and roots were particularly effective, already at 24 h of treatment, in reducing the cell viability of 42% and 95%, respectively, in HCT116 colon cancer cell line. These EOs also exerted a remarkable cytotoxic effect that was accompanied by morphological changes represented by cell shrinkage as well as a reduction in residual cell population. Differently, modest effects were found when EOs extracted from flowers were tested in the same experimental conditions. The evaluation of the phytocompounds mainly represented in the EOs extracted from different parts of the plant and tested in a range of concentrations between 20 and 200 μg/mL, revealed that *α*-pinene, *β*-pinene, and *p*-cymene exerted only modest effects on cell viability. Differently, a remarkable effect was found when *β*-ocimene, the most abundant phytocomponent in EOs from roots, was tested on colon cancer cells. This phytocompound, among those identified in EOs from *Seseli tortuosum* L. subsp. *tortuosum*, was found to be the most effective in reducing colon cancer cell viability with IC_50_ = 64.52 μg/mL at 24 h of treatment. All together, these data suggest that *β*-ocimene could be responsible for the effects observed in colon cancer cells.

## 1. Introduction

Natural compounds, isolable from plant and marine systems, have a long history in the treatment of several diseases [1]. Plants can provide a wide array of potential drugs, both natural and synthetically modified, demonstrating diverse biological effects [2,3].

Among the medicinal and aromatic plants, species of the Apiaceae family, once known as Umbelliferae or “Carrot Family” [4], are ethnobotanically among the most exploited in the plant world. It comprises 3800 species divided into 466 genera, and they are mainly distributed in the Mediterranean and Southwest Asia regions, generally in mild northern climates, high altitudes, and tropical areas. These plants find a crucial use in the culinary field (some are cooked and utilized as vegetables or as spices), and/or in pharmaceutical, cosmetic, and perfume sectors, having an important economic impact [5,6,7]. These applications are due to their schizocarp fruits containing oil ducts, from which most of the secondary metabolites, such as coumarins, terpenoids, and polyacetylenes, are obtained [8,9].

*Seseli* L., a term that comes from ancient Greek, is one of the largest genera (about 140 accepted species according to the POWO database [10]) of the Apiaceae family, and their species are widely distributed across Europe, Asia, Africa, North America, and Australia [11,12,13].

The *Seseli* genus belongs to the Spermatophyta division, Dicotyledones class, and Apiales order, and it is represented by intraspecific and interspecific diversity, 80 of which grow within Euro-Siberian and East Mediterranean phytogeographic regions. These plants are usually perennials or biennials and possess a unique fibrous collar at the base, distinguishing them from the other Apiaceae species and subspecies. The leaves are 1–4 pinnate, with or without braces; the bracteoles may be united or separated at the base, and the petals are usually yellow or purple. The fruits are typically oblong-ovoid, and the stylopodium is conical in shape, with permanent styluses. *Seseli* species and ssp. prefer arid conditions, and they are often found in rocky environments like limestone, cliffs, and rock crevices up to high altitudes (around 3000 m). They typically bloom from June to November, resulting in late fruit formation. However, many species remain poorly documented. One distinctive feature of *Seseli* species is their pungent and exotic unusual odor, primarily emanating from the stems and roots [5]. The continuous discovery of new species contributes to the biological diversity of the *Seseli* genus [13,14].

Plants of the *Seseli* genus have been used since ancient times in traditional medicine. For example, they have been used in Serbia, commonly named *Devesilje*, as a diuretic, tonic emmenagogue, and digestive remedies [15], in Ayurvedic medicine, mixed with the *Selinum* L. species, as a sedative for mental disorders [15,16], or in Pakistan, where *S. diffusum* (Roxb. ex Sm.) Santapau & Wagh is used as drugs for amenorrhea, rheumatism, and fever accompanied by cough.

The different parts of *Seseli* plants have also been used individually. For instance, the roots of *S. mairei* H. Wolff, known in China as *zhu ye fang feng*, have been used for human inflammation, pain, and edema problems [17], and also, the juice obtained from the roots of *S. libanotis* has been employed to treat joint pains [18,19]. The dried leaves of this plant have been used in animal nutrition, as a vegetable in Eastern Turkey, and to flavor and to preserve different cheeses [20,21]. On the other hand, the seeds of *S. indicum* Wight & Arn., *S. tortuosum* L., and *S. diffusum*, diffused in India and Turkey, have also been prescribed for their anthelmintic, emmenagogue, carminative, and antispasmodic properties [22,23,24].

Regarding the phytochemistry aspects, several bioactive compounds, especially coumarins and sesquiterpene lactones, are commonly present in *Seseli* species and are likely contributors to their biological and pharmacological effects [25]. Compounds such as khellactone esters, sesebrinol, sesebrin, sibiricol, osthenol, and meranzin hydrate were detected in the *S. sibiricum* root extract and are exclusive to *Seseli* species [26]; on the other hand, (+)-peucedanol and (+)-ulopterol were identified through an HPLC analysis in the *n*-hexane extract of *S. montanum* [27]; toruoside, instead, a coumarin glycoside, has been isolated from the aerial parts of *S. tortuosum* [28]. Finally, a simple prenylated-coumarin, umbelliprenin, was obtained from the chloroform extract of *S. annuum* aerial parts [29].

As concerns sesquiterpene derivatives, 2-farnesyl-6-methyl-benzoquinone, a chemotaxonomic marker of the *Seseli* genus, has been identified in ether extracts of *S. elatum* L. and *S. longifolium* L. [30]. In addition, from the non-polar extract of *S. vayredanum* aerial parts, 10-hydroxy-*α*-humulene, 10-angeloyloxy-*α*-humulene, and 2-epilazerine were isolated.

The chemical composition of some essential oils (EOs) from various *Seseli* plants has been extensively studied. These investigations revealed that the main components of these mixtures vary according to different species, pedoclimatic conditions, and geographical aspects.

For example, *α*-pinene and sabinene, compounds belonging to the monoterpene hydrocarbons class, and carotol, a sesquiterpenoid, are major components in the EOs of several species of this genus [31]. The composition of EOs can also differ significantly based on the geographical region and on the plant’s parts used. For example, the chemical composition of leaves, stems, flowers, and fruits EOs of *S*. *elatum* L., collected in the north-east of Italy, differed both quantitatively and qualitatively, highlighting the influence of pedoclimatic conditions and also showing compositional differences in the investigated vegetative parts [32,33].

Some *Seseli* plants, like *S. campestre* Besser and *S. globiferum* Vis., have peculiar components in their EOs, such as (*E*)-sesquilavandulol and its acetate derivate, which are not commonly found in other Apiaceae species [34,35]. From another investigation conducted on the EO of *S. libanotis* (moon carrot or mountain stone-parsley), it was observed how the chemical composition varied significantly between the different collection regions and the several vegetative parts, with a diverse abundance of terpenoids like *α*-pinene, sabinene, myrcene, and germacrene D [19,36,37]. Moreover, in the EOs of *S. rigidum* Waldst. & Kit., an endemic plant primarily found in Balkan countries, it was observed how the chemical composition was significantly diverse according to geographical conditions, climate, and plant parts used for the extraction [38,39].

In the EOs of species belonging to the Apiaceae family, monoterpene hydrocarbons are frequently the most abundant metabolites, where α-pinene is amongst the major compounds in many species, often in combination with other monoterpenes such as *β*-pinene and/or *p*-cymene. This feature has been reported for the following taxa: *Anthriscus nemorosa* (M.Bieb.) Spreng. [40], *Eryngium amethystinum* L. [41], *Smyrnium perfoliatum* L. [42], *Conopodium capillifolium* (Guss.) Boiss. [43], *Aegopodium podagraria* L. [44,45], *Athamanta turbith* (L.) Brot. ssp. *haynaldii* (Borbás & Uechtr.) TutinF [46], *Silaum silaus* (L.) Schinz & Thell. [47], *Physospermum cornubiense* (L.) DC [44], *Bupleurum fruticosum* L. [43], *Bupleurum rigidum* L. ssp. *paniculatum* (Brot.) H. Wolff [48], *Bupleurum gibraltaricum* Lam. [49], *Angelica archangelica* L. [50], *Ferulago campestris* (Besser) Grecescu [51], *Ferulago nodosa* (L.) Boiss. [52], *Peucedanum oreoselinum* (L.) Moench H C. [44], *Laserpitium gallicum* L. [53], *Laserpitium petrophilum* Boiss. et Heldr. [54], *Daucus carota* L. [55], and *Daucus reboudii* Coss. [56].

On the other hand, the occurrence of only *β*-pinene and/or *p*-cymene was reported in *Myrrhoides nodosa* (L.) [57], *Chaerophyllum aromaticum* L. [58], *Chaerophyllum hirsutum* L. [59], *Pimpinella saxifraga* L. [60], *Oenanthe crocata* L.H [61], *Oenanthe pimpinelloides* L.H [43], *Portenschlagiella ramosissima* Tutin [62], *Meum athamanticum* Jacq. [63], and *Prangos denticulata* Fisch et Mey. [64] species. Instead, the EOs of *Seseli* taxa are often characterized by the presence of large amounts of pinene derivatives such as in the species *Seseli campestre* Besser from Turkey (α-pinene 35.8%) [35], *Seseli montanum* L. from Greece (*α*-pinene 32.3%) [43], *Seseli peucedanoides* (Bieb.) Kos.-Pol. from Serbia (*α*-pinene 69.4%; *β*-pinene 4.9%) [65], *Seseli resinosum* Freyn et Sint. from Turkey (*β*-pinene 13.7%; *α*-pinene 13.7%) [66], *Seseli tortuosum* L. from Italy (*α*-pinene 18.6; *β*-pinene 13.2%) [67], and *Seseli tortuosum* L.H from Turkey (*α*-pinene 35.9%; *β*-pinene 7.0%) [68]. Consequently, these compounds can be considered a chemical marker of the *Seseli* genus.

Due to the wide range of biological activities, EOs derived from different *Seseli* species have been investigated for their cytotoxic effects on different cell lines, including macrophage cells and keratinocytes. These studies have reported significant cytotoxic properties. For instance, the cytotoxic activity of *S. petraeum* EO was observed to be remarkable against MCF-7 (human breast adenocarcinoma) and A549 (human lung carcinoma). This activity is attributed to carotol, a major component in this EO [69].

*Seseli tortuosum* EO exhibited cytotoxic effects against macrophage cells, with an IC_50_ ranging from 10 to 25 μg/mL [70]. Additionally, the EOs from *S. tortuosum* and *S. montanum* subsp. *peixotoanum* reduced the MTT reduction by keratinocytes, resulting in cytotoxicity effects. *Seseli tortuosum* EO was particularly cytotoxic, with a concentration of 0.64 μL/mL [71].

Moreover, *S. tortuosum* EO displayed promising antiproliferative properties in breast and colorectal cancer cell lines, inducing apoptosis and increasing p21 expression. The lowest IC_50_ value was recorded at 0.0086 μL/mL [72].

For this reason, considering the promising cytotoxic properties previously reported for the *Seseli* genus, particularly for *Seseli tortuosum*, the aim of this work is to investigate the chemical composition of EOs, extracted from different vegetative parts of *Seseli tortuosum* subsp. *tortuosum*, an endemic Sicilian plant, evaluating biological capacities and verifying how these can be related to the main and most abundant metabolites.

## 2. Results and Discussion

### 2.1. Chemical Composition of S. tortuosum subsp. tortuosum Essential Oils

*S. tortuosum* subsp. *tortuosum* EOs (**STT**), extracted from three different parts (flowers, stems, and roots) had a yellow straw color. Overall, twenty-three different compounds were identified, twelve in flowers’ EO (**FSTT**) (representing 96.61% of the total composition), twelve in stems’ EO (**SSTT**) (96.48%), and eleven in roots’ EO (**RSTT**) (92.07%). All identified compounds are listed in Table 1.

The three EOs, although presenting a similar percentage of the principal main class (monoterpene hydrocarbons), were found to be very dissimilar in their chemical compositions. **FSTT**, compared to the other two, was found to be less abundant in monoterpene hydrocarbons class (79.98%), with *p*-cymene (31.83%), *α*-pinene (11.43%), *β*-pinene (9.29%), and *α*-terpinene (9.25%), as the main class’s constituents, followed by *γ*-elemene (4.94%, sesquiterpene hydrocarbon), farnesol (6.01%, oxygenated sesquiterpene), and anisole (5.05%) for the oxygenated monoterpenes class.

The **SSTT** showed a higher abundance of monoterpene hydrocarbons (91.21%), with 3-carene (19.71%), *β*-pinene (19.32%), sylvestrene (17.20%), and *α*-pinene (16.36%) as metabolites present in greater quantities, while the second most abundant class was that of the oxygenated monoterpenes (4.56%), with thymol methyl ether (3.41%) as the principal compound.

Finally, **RSTT** differed from the two previous EOs mentioned, showing similar percentage of monoterpene hydrocarbons (87.94%) but with a different abundance of *α*-pinene (32.98%), *β*-pinene (13.65%), and 3-carene (11.57%), containing instead *β*-ocimene (16.29%) and *allo*-ocimene (6.58%), not found in the other EOs.

The data obtained agreed with the previous paper where only the chemical composition of the *S. tortuosum* subsp. *tortuosum* aerial parts’ EO was investigated, in which the main components were *β*-pinene (15.81%), *α*-pinene (14.63%), 3-carene (14.58%), sylvestrene (11.18%), and *p*-cymene (11.14%), and with the absence of *β*-ocimene and *allo*-ocimene, principal compounds of the **RSTT** sample [73].

The scientific literature does not offer results on the EO composition of *S. tortuosum* ssp. species, except for *S. tortuosum* subsp. *maritimum*. In fact, flowers’ EO showed, as the main class, the monoterpene hydrocarbons (51.91%), with *p*-cymene (24.32%), *α*-pinene (13.67%), and sylvestrene (13.38%) as major components, absent in **FSTT** composition. Instead, stems’ and roots’ EOs presented a similar chemical composition with the occurrence of *α*-pinene, *β*-pinene, sylvestrene, *β*-ocimene, and *allo*-ocimene as principal metabolites. The only difference was the absence of 3-carene in both **SSTT** and **RSTT** EOs [74].

As regards other investigations on *S. tortuosum* L. EOs, the literature only offers studies carried out on volatile extracts obtained from the aerial parts only, not taking into consideration the different vegetative parts, which therefore cannot be compared exactly with the data of this present work. Despite this, it is possible to describe some conclusions, such as the fact that the aerial parts’ EO of *S. tortuosum,* collected in the Iran regions, contained a significant quantity of *β*-phellandrene (14.9%), a compound absent in **FSTT** and **SSTT** samples [75]. It is also interesting to point out, that **STT** is also different from *S. tortuosum* examples, collected always in Turkey, which had a greater quantity of *α*-pinene (35.90%) and *trans*-sesquilavandulol (8.40%) [67]. It turned out differently than one collected in Pisa, Italy, characterized by the presence of myrcene (29.20%) and acorenone (6.30%) as the majority metabolite [68], not present in our EOs of flowers and stems.

However, the data obtained additionally showed the difference between **STT** and the other two Sicilian endemic *Seseli* species, *S. tortuosum* subsp. *maritimum,* discussed previously, and *S. bocconei*, which had an almost equal distribution of monoterpene (37.95%) and sesquiterpene hydrocarbons (53.27%). This last composition, also positively influenced by the moderate presence of germacrene D (24.53%) as the majority metabolite, differed markedly from these results reported here [76].

### 2.2. Effects of S. tortuosum subsp. tortuosum EOs on Tumor Cell Viability

**Figure 1:**
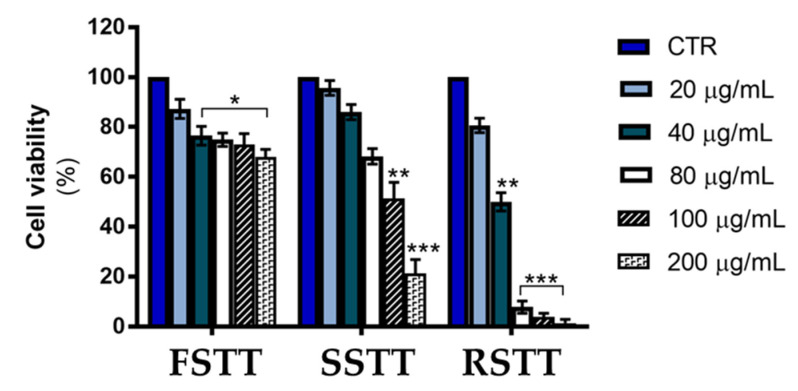
Cytotoxic effects exerted by **FSTT**, **SSTT**, and **RSTT** in HCT116 colon cancer cells. The cell viability was analyzed after 24 h of treatment with different doses of different EOs. Data reported in the bar chart are expressed as the means ± SD of experiments performed in triplicate. * *p* < 0.05, ** *p* < 0.01 and *** *p* < 0.001 compared to the untreated cells.

To ascertain whether EOs of *S. tortuosum* subsp. *tortuosum* are capable of exerting antiproliferative activity in tumor cells, MTT cell viability assay was performed on HCT116 cells, a human colon cancer cell line. To this purpose, HCT116 cells were treated for 24 h with different doses (range 20–200 μg/mL) of **FSTT**, **RSTT**, and **SSTT** before proceeding with cell viability analysis. Data reported in Figure 1 provided evidence that all EOs reduced cell viability of colon cancer cells in a dose-dependent manner. However, by comparing the effects of different EOs, it was observed that **SSTT** and **RSTT** exerted a remarkable effect in reducing cell viability of HCT116 cells, with **RSTT** emerging as the most effective, while only modest effects were observed with **FSTT**. Indeed, at the dose of 100 μg/mL, the viability of HCT116 cells was reduced by 95% with **RSTT**, by 42% with EOs of **SSTT**, and by only 22% with **FSTT**.

**Figure 2:**
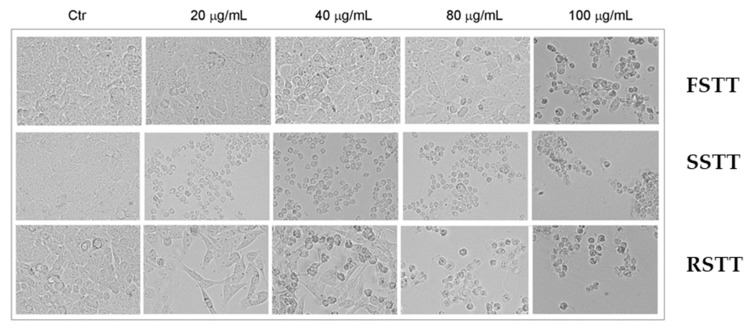
Morphological effects exerted by EOs prepared from **FSTT**, **SSTT**, and **RSTT** in HCT116 colon cancer cells. After incubation with EOs, pictures were taken at 200× magnification by the OPTIKA IM3FL4 microscope equipped with a digital imaging camera system (OPTIKA, Ponteranica, Italy).

These data were confirmed by microscope images showing that the treatment of tumor cells with EOs from stems and roots was accompanied by morphological changes represented by cell shrinkage as well as a cell number reduction (Figure 2).

**Figure 3:**
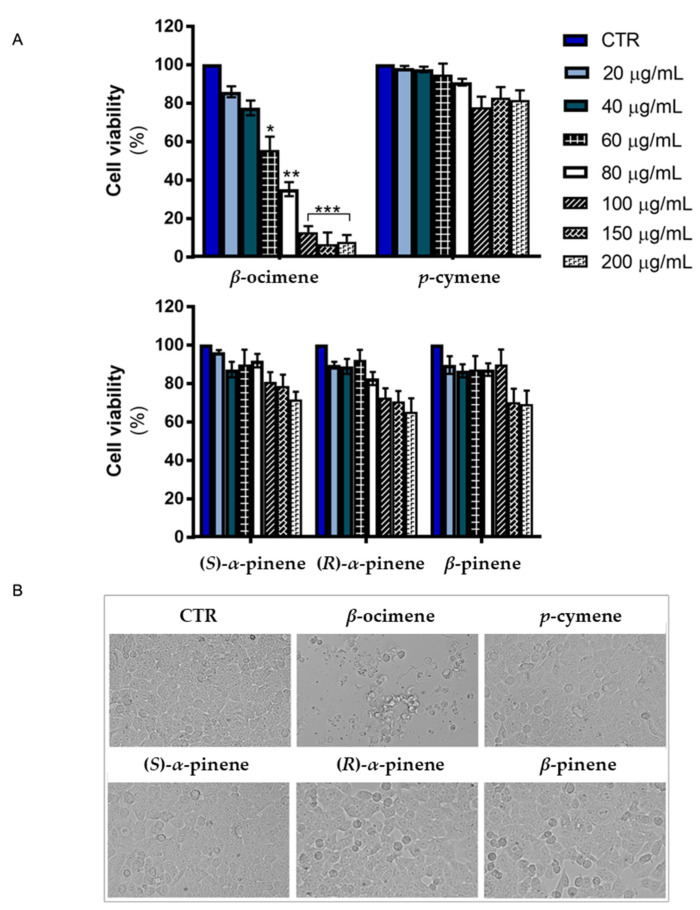
Analysis of the impact of the major phytocompounds identified in the **STT** EOs on HCT116 colon cancer cell viability. (**A**) HCT116 colon cancer cells were treated with the indicated compounds for 24 h, then cell viability was evaluated by MTT assay as reported in methods. Data reported in the bar chart are expressed as the means ± SD of experiments performed in triplicate. * *p* < 0.05, ** *p* < 0.01 and *** *p* < 0.001 compared to the untreated condition; (**B**) Morphological effects exerted by 100 μg/mL dose of the main phytocompounds (*β*-ocimene, *p*-cymene, (*S*)-*α*-pinene, (*R*)-*α*-pinene, and *β*-pinene) of the EOs in HCT116 colon cancer cells. The pictures were taken at 200× magnification by the OPTIKA IM3FL4 microscope.

To clarify which compounds could be responsible for the cytotoxic effects exerted by **STT** EOs, the impact of their major components in EOs on colon cancer cell viability (Figure 3A) was evaluated. Comparing the effect of different doses (20–200 μg/mL) of each compound on cell viability of HCT116 colon cancer cells, the most significant cytotoxic effect was observed when HCT116 cells were incubated in the presence of β-ocimene. Indeed, calculating the half-maximal drug inhibitory concentration (IC_50_ value) for this compound on colon cancer cell viability, it was found that it amounted to IC_50_ = 64.52 μg/mL at 24 h of treatment. Such an effect was also confirmed by morphological analyses performed under light microscopy and shown in Figure 3B. In β-ocimene-treated cells, a clear cytotoxic effect was observed. Indeed, the incubation in the presence of β-ocimene caused a reduction in cell number as well as cell shrinkage, two typical hallmarks of programmed cell death. Differently, when the other compounds, p-cymene, (S)-α-pinene, (R)-α-pinene, and β-pinene, were tested on HCT116 colon cancer cell viability, no significant cytotoxic effect was observed.

## 3. Materials and Methods

### 3.1. Plant Material

Flowers, stems, and roots from several fresh individuals of *S. tortuosum* subsp. *tortuosum*, at the full flowering stage, were collected at Mojo Alcantara, Sicily, Italy, on volcanic substrate at about 640 m a.s.l., 37°54′35.90′′ longitude N and 15°02′55.57′′ latitude E, in December 2021. One of the samples, identified by Prof. Vincenzo Ilardi, has been stored in the University of Palermo Herbarium (No. PAL 109751).

### 3.2. Essential Oils Extraction

Extraction of EOs was performed according to Vaglica et al. [77]. Fresh flowers (103.00 g), stems (329.00 g), and roots (80.00 g) were separately hand-cut into small fragments, and then subjected to hydrodistillation for 3 h, according to the standard procedure described in European Pharmacopoeia (2020) [78]. Samples yielded 0.26%, 0.15%, and 0.13%, for **FSTT**, **SSTT**, and **RSTT**, respectively.

### 3.3. GC-MS Analyses

Analysis of EOs was carried out according to the procedure reported by Castigliuolo et al. [79]. Linear retention indices (LRIs) were calculated using a mixture of pure *n*-alkanes (C_8_–C_40_), and all the peaks’ compounds were identified by comparison with MS and by comparison of their relative retention indices with WILEY275, NIST 17, ADAMS, and FFNSC2 libraries.

### 3.4. Pure Compounds

All pure compounds, such as α-pinene, β-pinene, and β-ocimene were purchased from Sigma-Aldrich (Sigma-Aldrich Chemie GmbH, Eschenstr. 5, 82024 Taufkirchen, Germany).

### 3.5. Cell Culture

HCT116 human colon cancer cells were purchased from Interlab Cell Line Collection, ICLC, Genova, Italy. Cells were cultured in flasks of 75 cm^2^ in RPMI 1640 medium, supplemented with 10% (*v*/*v*) heat-inactivated fetal bovine serum (FBS), 2 mM L-glutamine, 100 U/mL penicillin, and 50 μg/mL streptomycin in a humidified atmosphere of 5% CO_2_ in air at 37 °C [80]. For this study, before treatments, cells were detached by trypsin-EDTA solution (0.5 mg/mL trypsin and 0.2 mg/mL EDTA) and seeded in 96-wells plates as reported below. All stock solutions of EOs from **STT**, as well as the single compounds tested, were prepared in DMSO and stored at −20 °C until use. In each experiment, working solutions were solubilized in RPMI medium, never exceeding the DMSO volume percentage of 0.01% (*v*/*v*). The vehicle condition represented by untreated cells incubated in the presence of the corresponding DMSO volume was reported as control.

### 3.6. Analysis of Cell Viability and Morphological Effects

To evaluate the effects of different parts of EOs of **STT** as well as the action of major phytocompounds identified, HCT116 cells were plated on 96 wells at a density of 7 × 10^3^/200 μL/well, and then, were incubated overnight at 37 °C. After 24 h, cells were treated with different doses of all EOs or major compounds, and the incubation was protracted for 24 h. Then, the viability was assayed by MTT [3-(4,5-dimethylthiazol-2-yl)-2,5- diphenyl tetrazolium bromide] colorimetric assay as previously reported [81,82]. This assay estimates the metabolic activity of living cells by reduction of MTT into formazan by mitochondrial dehydrogenases. In the end, the absorbance of the formazan was measured directly at 490 nm with 630 nm as a reference wavelength using an automatic ELISA plate reader (OPSYS MR, Dynex Technologies, Chantilly, VA, USA). Cell viability was expressed as the percentage of the OD value of EO-treated cells compared with untreated samples used as control (100% viability). Each experiment was performed in triplicate, and data are the mean ± SD of three independent experiments. The IC_50_ values were performed using Graphpad Prism 7.0 software (San Diego, CA, USA) as previously reported [83]. The analysis of morphological effects induced by treatments was performed by an OPTIKA IM3FL4 microscope equipped with a digital imaging camera system (OPTIKA). The pictures were captured by the OPTIKA PROVIEW software (version x64, 4.11.20805.20220506).

### 3.7. Statistics

All data were reported as mean ± S.D., and analyses were performed using the Student’s *t*-test and one-way analysis of variance by Graphpad Prism 7.0 software (San Diego, CA, USA). Comparisons between the control (untreated) and all treated samples were made. The statistical significance threshold was fixed at *p* < 0.05.

## 4. Conclusions

In this study, the chemical profile of EOs of *Seseli tortuosum* L. subsp. *tortuosum* in its different vegetative parts, flowers, stems, and roots was examined. The most abundant class, for all EOs examined, was that of monoterpene hydrocarbons, in which the main compounds were *p*-cymene, *α*-pinene, *β*-pinene, sylvestrene, 3-carene, and *β*-ocimene. These compositions were found to be different from the other two Sicilian *Seseli* accessions, *Seseli bocconei* and *Seseli tortuosum* subsp. *maritimum*, not only in terms of the main components but also in the different sub-division into metabolite classes. To explore the anticancer potential of the three EOs as well as their main components, we tested their effects on colon cancer cells. Interestingly, reported data clearly evidenced that, among the different EOs tested, those extracted from stems and roots could induce remarkable cytotoxic effects on colon cancer cells. However, following our previous studies [74], *α*-pinene or *β*-pinene, some of the main phytocompounds of EOs, exerted only modest effects in colon cancer cells. Differently, significant effects were found in the presence of *β*-ocimene. Indeed, this constituent resulted in being the most effective in reducing cell viability with an IC_50_ equal to 64.52 μg/mL at 24 h of treatment, producing both cell number and volume reduction, two known hallmarks of programmed cell death.

## Figures and Tables

**Table 1:** Chemical composition of **FSTT**, **SSTT**, and **RSTT** essential oils (EOs).

No.	Compounds ^a^	LRI ^b^	LRI ^c^	FSTT ^d^	SSTT ^d^	RSTT ^d^
**1**	*α*-Pinene ^e^	1005	1007	11.43	16.36	32.98
**2**	Camphene	1026	1037	-	5.04	-
**3**	*β*-Pinene ^e^	1079	1091	9.29	19.32	13.65
**4**	Sabinene	1105	1109	6.92	7.69	-
**5**	3-Carene	1157	1158	5.05	19.71	11.57
**6**	*β*-Myrcene ^e^	1174	1176	2.44	5.53	2.82
**7**	*α*-Terpinene	1183	1190	9.25	-	-
**8**	Limonene ^e^	1185	1191	3.77	-	-
**9**	Sylvestrene	1192	1202	-	17.20	4.05
**10**	*p*-Cymene	1245	1248	31.83	-	-
**11**	*β*-Ocimene	1241	1246	-	-	16.29
**12**	Allo-Ocimene	1377	1382	-	0.36	6.58
**13**	Fenchyl acetate	1478	1481	-	-	0.66
**14**	Pinocarvone	1567	1575	-	0.34	-
**15**	Isobornyl acetate	1570	1574	-	-	-
**16**	4-Terpineol	1581	1585	-	0.81	-
**17**	Anisole	1592	1595	5.05	-	-
**18**	Thymol methyl ether	1601	1607	-	3.41	-
**19**	*γ*-Elemene	1645	1650	4.94	-	0.79
**20**	Germacrene D	1696	1703	-	0.71	2.10
**21**	Octanoic acid	2073	2073	-	-	0.58
**22**	Farnesyl acetate	2213	2220	0.63	-	-
**23**	Farnesol	2316	2318	6.01	-	-
	**Monoterpene Hydrocarbons**			**79.98**	**91.21**	**87.94**
	**Oxygenated Monoterpenes**			**5.05**	**4.56**	**0.66**
	**Sesquiterpene Hydrocarbons**			**4.94**	**0.71**	**2.89**
	**Oxygenated Sesquiterpenes**			**6.64**	**-**	**-**
	**Other Compounds**			**-**	**-**	**0.58**
	**Total**			**96.61**	**96.48**	**92.07**

^a^ Components listed in order of elution on a DB-Wax column; ^b^ Linear retention indices on a DB-Wax polar column; ^c^ Linear retention indices based on literature (https://webbook.nist.gov/ accessed on 10 January 2024); ^d^ Percentage amounts of the separated compounds calculated from integration of the peaks; ^e^ Co-injection with authentic standards.

## Data Availability

All data and materials are available upon request from the corresponding author.
